# Prolonged Calcitonin Receptor Signaling by Salmon, but Not Human Calcitonin, Reveals Ligand Bias

**DOI:** 10.1371/journal.pone.0092042

**Published:** 2014-03-18

**Authors:** Kim Vietz Andreassen, Sara Toftegaard Hjuler, Sebastian G. Furness, Patrick M. Sexton, Arthur Christopoulos, Olivier Nosjean, Morten Asser Karsdal, Kim Henriksen

**Affiliations:** 1 Nordic Bioscience A/S, Herlev, Denmark; 2 Drug Discovery Biology and Department of Pharmacology, Monash Institute of Pharmaceutical Sciences, Monash University, Victoria, Australia; 3 Institut de Recherches Servier, Croissy-sur-Seine, France; Medical School of Hannover, Germany

## Abstract

Salmon calcitonin (sCT) and human calcitonin (hCT) are pharmacologically distinct. However, the reason for the differences is unclear. Here we analyze the differences between sCT and hCT on the human calcitonin receptor (CT_(a)_R) with respect to activation of cAMP signaling, β-arrestin recruitment, ligand binding kinetics and internalization. The study was conducted using mammalian cell lines heterologously expressing the human CT_(a)_ receptor. CT_(a)_R downstream signaling was investigated with dose response profiles for cAMP production and β-arrestin recruitment for sCT and hCT during short term (<2 hours) and prolonged (up to 72 hours) stimulation. CT_(a)_R kinetics and internalization was investigated with radio-labeled sCT and hCT ligands on cultured cells and isolated membrane preparations from the same cell line. We found that sCT and hCT are equipotent during short-term stimulations with differences manifesting themselves only during long-term stimulation with sCT inducing a prolonged activation up to 72 hours, while hCT loses activity markedly earlier. The prolonged sCT stimulation of both cAMP accumulation and β-arrestin recruitment was attenuated, but not abrogated by acid wash, suggesting a role for sCT activated internalized receptors. We have demonstrated a novel phenomenon, namely that two distinct CT_(a)_R downstream signaling activation patterns are activated by two related ligands, thereby highlighting qualitatively different signaling responses in vitro that could have implications for sCT use in vivo.

## Introduction

The calcitonins (CT) are a group of evolutionary conserved 32 amino acid peptide hormones, which have been isolated from many different species over the last 50 years and divided into several distinct groups based on structure and sequence [1]. The calcitonins bind to the calcitonin receptor (CT_(a)_R), which belongs to the class B subfamily of G-protein coupled receptors (GPCRs) [2], [3]. The CT_(a)_ and CT_(b)_ receptors are the two major alternatively spliced isoforms expressed in humans and have different properties with respect to activation of downstream signaling pathways for each isoform and in their tissue distribution, with the CT_(a)_ receptor being the most abundant [3], [4]. The CT_(a)_ receptor mediates downstream signaling through most of the classic GPCR pathways including cAMP induction, β-arrestin recruitment, calcium mobilization and ERK activation [5]–[9]. The teleost/avian group of calcitonin peptides is the most potent of the calcitonin groups, and is exemplified by salmon calcitonin (sCT), which was the first high potency calcitonin to be discovered and this peptide is used as the benchmark in estimations of calcitonin potency from other species [1]. In general, any increased homology with sCT increases the biological activity of the agonists [10]. The teleost/avian calcitonins are a unique group among the calcitonins as they bind with high affinity and potency to the CT_(a)_R [1] as well as to the amylin receptor (AMY-R) with sCT being a more potent agonist compared to amylin [11]. sCT binds almost irreversibly, with a very slow off rate from the CT_(a)_R [12] and induces a sustained cAMP accumulation [3], [13], [14]. In comparison, following short term simulation human calcitonin (hCT) is equipotent to sCT with respect to CT_(a)_R activation *in vitro*, while hCT has poor affinity for AMY-Rs [11], [12]. The high potency of sCT made it a target for drug development for treatment of osteoporosis and osteoarthritis [4], [15]. Furthermore, sCT has recently been suggested as a potential treatment of type II diabetes mellitus [16], [17]. sCT is much more potent *in vivo* [1], and this has assumptively been associated with longer plasma half-life. However, this clear cut difference is somewhat blurred as this is not always reflected in comparable *in vitro* studies [3], [12]–[14], [18]–[20]. It is apparent that sCT is different from hCT, as they only share 50% sequence homology, see Table 1, but whether this difference is purely a consequence of activation potency or whether other mechanisms are involved remains to be elucidated.

**Table 1:** Amino acid sequence comparison of salmon and human calcitonin.

Residue	1	2	3	4	5	6	7	8	9	10	11	12	13	14	15	16	17	18	19	20	21	22	23	24	25	26	27	28	29	30	31	32	
Salmon Calcitonin	C	*S*	N	L	S	T	C	**V**	L	G	**K**	**L**	**S**	Q	*E*	**L**	*H*	K	**L**	*Q*	T	*Y*	P	**R**	T	**N**	**T**	G	**S**	G	**T**	P	-NH2
Human Calcitonin	C	*G*	N	L	S	T	C	**M**	L	G	**T**	**Y**	**T**	Q	*D*	**F**	*N*	K	**F**	*H*	T	*F*	P	**Q**	T	**A**	**I**	G	**V**	G	**A**	P	-NH2

*Italic, underlined* amino acids illustrate conservative substitutions. Amino acids in **bold** illustrate non-conservative substitutions. Disulfide bridge between C1-C7.

In this paper, we used cells heterologously expressing the CT_(a)_ receptor to perform a head-to-head comparison of hCT and sCT focused on elucidating any differences in the ability to bind, activate and introduce down-regulation of the receptor as a function of ligand concentration and more importantly exposure time.

## Methods

### Calcitonin ligands

sCT (H-2260, Bachem), hCT (H-2250, Bachem), ^125^I-(Tyr22)-sCT (NEX423, Perkin Elmer) and ^125^I-(Tyr12)-hCT (NEX422, Perkin Elmer). *U2OS CALCR and COS-7 1375 cell lines:* U2OS CALCR cells (PathHunter U2OS CALCR β-arrestin cell line, DiscoverX: 93-0566C3) cultured in MEM Eagle 1× (30-2003, ATCC) supplemented with 10% Fetal Bovine Serum (FBS) (F2442, Sigma-Aldrich), 1% Penicillin-streptomycin (P/S) (10376-016, Invitrogen), 300 μg/mL Hygromycin B (10687-010, Invitrogen) and 800 μg/mL Geneticin (10131-019, Invitrogen).

*Cos-7 CT*_(a)_ *R cell line*: parental Cos-7 cell line (CRL-1651, ATCC) with a stable transfection of a pEF-IRESpuro6 expression vector [21] containing a cDNA insert for a cMyc-hCT_(a)_(Leu variant) receptor construct (The stable transfected Cos-7 cell line was provided by Monash Institute of Pharmaceutical Sciences, DBB number: Cos-7 1375.10). The Cos-7 CT_(a)_R cells were cultured in DMEM (21063-029, Invitrogen) supplemented with 5% FBS, 1% P/S and 10 μg/mL Puromycin (P8833, Sigma-Aldrich). All live cells experiments were incubated at 37°C in a humidified incubator with atmospheric air supplemented with 5% CO_2_ unless otherwise specified.

### β-arrestin and cAMP quantification

Both assays were performed in white 384 well plates (784080, Greiner Bio-One). Appropriate cells were seeded at a concentration of 2,500 cells/well in 10 μL cell-type specific medium the day prior to the experiment. Quantification of the CT_(a)_R-mediated β-arrestin recruitment in U2OS CALCR cells was measured directly in the wells with the PathHunter Detection Kit [22] (93-0001, DiscoverX) according to the manufacturer's instructions. Plates were incubated with detection reagents for 60 mins at RT, and determined the relative luminescence signal (RLU) using a SpectraMax M5 Multimode Plate Reader (Molecular Devices).

cAMP assays were conducted with 100 μM IBMX for short term stimulation (Total production) or without IBMX for prolonged responses (Time dependent accumulation) in both U2OS CALCR and COS-7 CT_(a)_R cells. Quantification of the intracellular cAMP was measured directly in the wells using the cAMP FEMTO TB KIT [23] (62AM7PEB, Cisbio Bioassays) according to the manufacturer's instructions. Plates were incubated with detection reagents for 60 min at 4°C and signal determined using a VICTOR 3 Multilabel Counter Model 1420 (Perkin Elmer). For the cAMP assay, cell protein content was determined using the DC Protein Assay Kit II (Cat.no.:500-0111, BioRad, USA).

### Acid wash

#### cAMP and β-arrestin

2,500 U2OS CALCR cells/well were incubated with ligands in a standard β-arrestin setup for 60 min. Medium was aspirated and cells washed two times for 2 min in Acid Buffer (150 mM NaCl, 50 mM glycine, pH = 2.5 as described by Michelangeli *et al* [13] followed by two additional washes in fresh culture medium to remove residual acid. Cells were then incubated for the remaining incubation time as described in the results section.

#### Receptor internalization

15,000 U2OS CALCR cells/well were seeded overnight, and left at 4°C for 45 min prior to experiment. Cells were then incubated with either ^125^I-(Tyr 22)-sCT and ^125^I-(Tyr 12)-hCT for 45 min at 4°C to attenuate internalization during ligand binding. Internalization was initiated by raising temperature to 37°C using a humidified incubator with atmospheric air supplemented with 5% CO_2_. Internalization was assessed at time 0, 15, 30, 60 and 120 min by washing twice for two min with either PBS wash (Total binding) or acid wash (Internalized fraction). After the wash, cells were lysed with 200 μL 0.5 M NaOH, and allowed to solubilize for at least 20 min. Lysate ^125^I-sCT or ^125^I-hCT content was measured using a 1470 Wallac Wizard Gamma Counter.

### Membrane-based Receptor Binding Kinetics

The binding kinetics of ^125^I-(Tyr 22)-sCT and ^125^I-(Tyr 12)-hCT were assessed using isolated membranes from Cos-7 CT_(a)_R or U2OS CALCR. Concentration of radio-labeled ligand was 0.25 nM unless otherwise specified. Membranes were isolated and purified as previously described [24]. Membrane protein content was determined with a NanoDrop 1000 (Thermo Scientific). In general, radio-labeled ligand and membrane were mixed in binding buffer (20 mM HEPES, 1 mM CaCl_2_, 5 mM MgCl_2_) with complete protease inhibitor (11836170001, Roche), each condition in triplicate, in 96-well plates and incubation at room temperature (∼22°C) with shaking. *Ligand association:* The radio-labeled ligand was incubated with the membrane and the membrane-bound radioactivity was determined as described above at specific time points 2½, 5, 10, 20, 40, 80 and 160 min. Nonspecific binding was determined at each time point with co-incubation of 250 nM unlabeled sCT. *Ligand dissociation:* The radio-labeled ligand and membrane were incubated for one hour to allow ligand-receptor saturation. Dissociation was monitored following the addition of 250 nM unlabeled sCT to prevent rebinding of dissociated radio-ligand. The membrane-bound radioactivity (total binding) was determined at time 0, 1, 2, 4, 6, 24, 48 and 72 h after the initiation of dissociation. *Competitive ligand binding:* The radio-labeled ligand and membrane were co-incubated with one of seven different concentrations of corresponding unlabeled ligand (1 μM sCT or hCT with a fivefold dilution row) for one hour. *Post incubation sample extraction:* The binding mixtures were transferred to 1.5 ml Eppendorf tubes and centrifuged at 17,000 g (4°C for 5 min). Membrane pellets were washed with cold binding buffer, resuspended and then re-centrifuged (17,000 g, 4°C for 5 min).

### Cell-based Receptor Binding Kinetics/Ligand processing

#### Association, dissociation and competitive binding

15,000 U2OS CALCR cells/well were seeded into 96 well culture plates the day prior to the experiment. All cell based binding kinetics studies were made identical in terms of ‘hot’ and ‘cold’ ligand concentrations and incubation times to match membrane studies. *Post incubation sample extraction:* The binding mixture and cells were mixed in culture medium in triplicates in 96-well plates and incubated at 37°C. At the end of the incubation, the binding mixture was removed and cells were washed three times in PBS to remove residual unbound ligand. 200 μL 0.5 M NaOH was added to each well and cells were allowed to solubilize for at least 20 min.

### Quantification of ^125^I bound ligand activity

Samples, resuspended pellet or solubilized cell membrane, were transferred to borosilicate tubes and bound radioactivity was measured using a 1470 Wallac Wizard Gamma Counter.

### ERK1/2 western blotting

Cos-7 CT_(a)_R cells was cultured in 75 cm^2^ culture flasks to 90% confluency and treated with 100 nM hCT, 100 nM sCT or vehicle for 5 min, 10 min, 30 min, 120 min, 24 hours or 48 hours. At each time point the cells were washed in PBS and then lysed. *Lysate preparation:* Cells were lysed using RIPA‡ buffer (30 mM NaCl, 50 mM Tris-HCl, 5 mM EDTA,1% Deoxycholic acid. 10% SDS) and cleared by centrifugation (20 min, 20.000 g) and protein concentration was determined by using DC Protein Assay Kit II (Cat.no.:500-0111, BioRad, USA). *Electrophoresis and blotting:* 1 μl of NuPAGE Reducing agent and 2.5 μl NuPAGE LDS 4× Sample buffer were added to 20 μg sample and volume adjusted to 10 μl with MilliQ H2O. Samples were boiled at 70°C for 10 min and loaded onto a NuPAGE 10% Bis-Tris Mini Gel (NP0301BOX, Invitrogen) and run at 150 V for 1 hour, then blotted onto a nitrocellulose membrane at 60 mA for 2 hours. The membrane was initially probed with the Phospho-ERK1/2 antibody (1∶2000 dilution, #9106, Cell Signaling Technology) using HRP-rabbit-anti-mouse (1∶5000 dilution, 315-035-045, Jackson ImmunoResearch Laboratories, Inc.) as secondary antibody. After visualization with ECL kit (#32209, Thermo Scientific) the membrane was stripped and re-probed with the total ERK1/2 (1∶1000 dilution, #4695, Cell Signaling Technology) using HRP-goat anti-rabbit (111-035-144, Jackson ImmunoResearch Laboratories, Inc.) as secondary antibody. Total ERK1/2 was used as a protein loading control.

### Data and Statistical Analysis

All data and statistical analyses were performed using GraphPad Prism software (GraphPad Prism 5.03, San Diego, CA. U.S.A). All data are presented as mean ± standard error of the mean (SEM). β-arrestin data are shown as mean ± SEM of three experiments with five replicates. cAMP data is illustrated as a single experiment that are representative of a series of three experiments. The statistical analysis of vehicle effects versus sCT or hCT effects were analyzed by one-way ANOVA followed by the Bonferroni selected pair post hoc test. Alternatively the Student's t-test was used to compare sCT and hCT groups directly. For complete dose response assessment, total area under the curve (tAUC) was approximated using the AUC trapezoidal method and AUC values were analyzed by an unpaired, two-sided Student's t-test. A value of p<0.05 was considered to be significant. *  = p<0.05, **  = p<0.01, ***  = p<0.001.

## Results

### sCT and hCT show highly similar short-term receptor binding and activation properties

**Figure 1:**
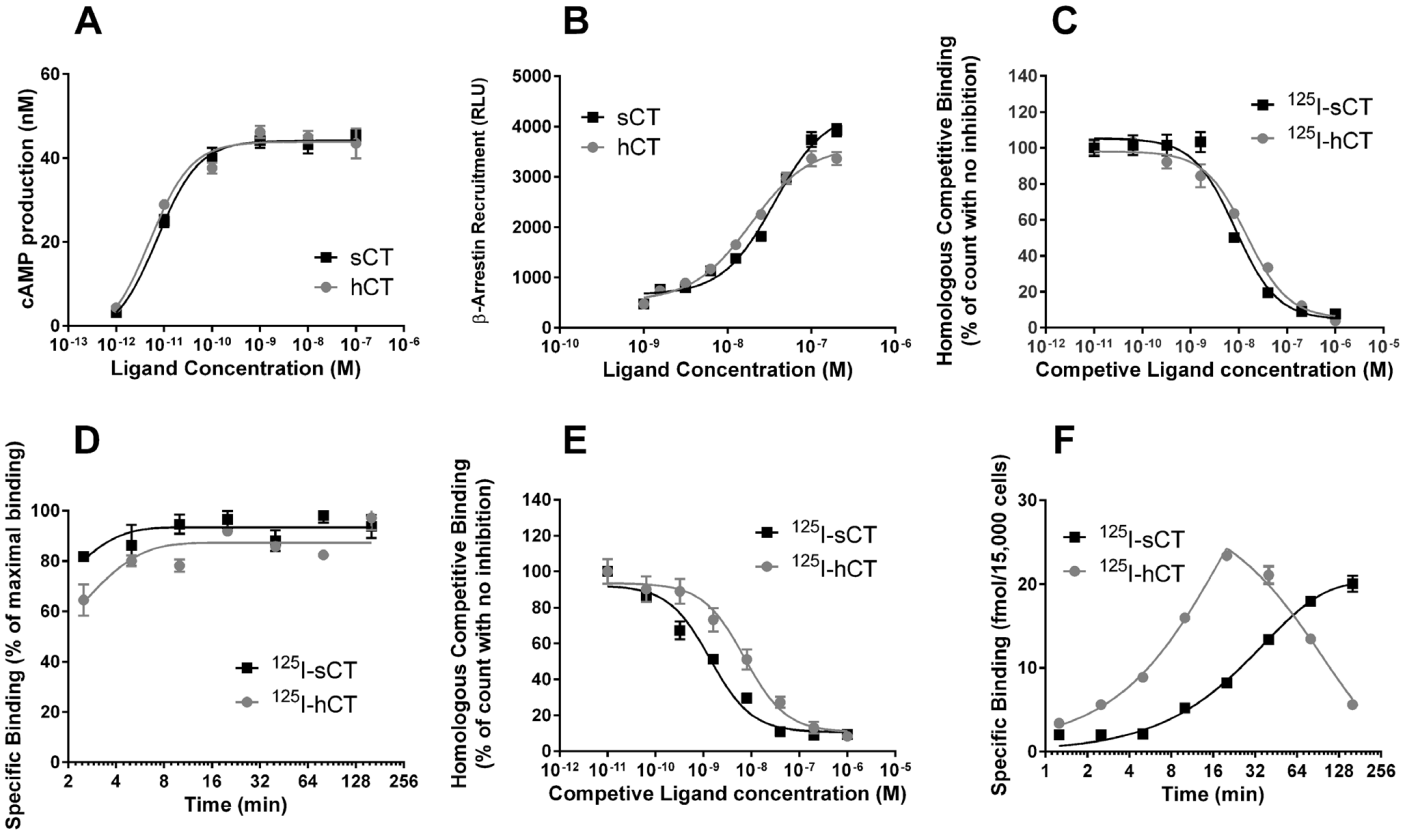
Functional output of cAMP, β-arrestin, ligand binding on membranes and live cells in response to calcitonins. Head-to-head comparison of dose dependent cAMP accumulation (A) or β-arrestin recruitment (B), mediated by sCT and hCT after one (cAMP assay) and two (β-arrestin assay) hours of stimulation, respectively. The cAMP experiment was conducted with 100 μM IBMX in the medium to assess total cAMP production. Homologous competitive binding (C) and Ligand association (D) profiles of sCT and hCT using U2OS CALCR membranes preparations. Homologous competitive binding (E) and association (F) profiles of sCT and hCT using U2OS CALCR cells in culture. Data are shown as mean ± SEM and representative of three individual conducted experiments with six replicates (A,B) or three replicates (C–F).

sCT and hCT were tested at one hour stimulation for cAMP with 100 μM IBMX in the medium (Figure 1A) and at two hours ligand stimulation for β-arrestin recruitment (Figure 1B). Both assays demonstrated sCT and hCT to be equipotent within this short time frame. For cAMP production the calculated EC_50_ was 7.2±1.2×10^−12^ M and 5.0±1.3×10^−12^ M for sCT and hCT, respectively. The corresponding values for β-arrestin recruitment were 3.6±1.1×10^−8^ M and 1.9±1.1×10^−8^ M. These data also show that a markedly higher ligand concentration was needed to induce β-arrestin recruitment than cAMP accumulation; however, this was independent of ligand type. We assessed the association and competitive binding of sCT and hCT on isolated membranes, respectively. Specific binding of ^125^I-sCT and ^125^I-hCT to isolated membranes was very rapid and approached a plateau after ∼8 min for both ligands with association constants, K_ON_, of 3.1±0.5×10^9^ M^−1^min^−1^ and 2.1±0.3×10^9^ M^−1^min^−1^, respectively (Figure 1D). The corresponding IC_50_ values determined by homologous competitive binding on membranes were 8.5±1.2×10^−9^ M and 14.3±1.2×10^−9^ M, respectively (Figure 1C). Specific binding of ^125^I-sCT to live cells approached a plateau after 180 min with a K_ON_ of 1.1±0.1×10^8^ M^−1^min^−1^ (Figure 1F). The corresponding IC_50_ value for sCT in homologous competition of ^125^I-sCT binding to live cells was 1.4±1.2×10^−9^ M (Figure 1E). Interestingly, specific binding of ^125^I-hCT to live cells did not follow a classical association curve. Maximal binding was observed after 20 min, after which the level of specific binding declined leaving <20% bound after 180 min when compared to 20 min (Figure 1F); hence no K_ON_ value was calculated. Peak binding for both ^125^I-hCT and ^125^I-sCT, however, was similar. The corresponding IC_50_ value for hCT in homologous competition for ^125^I-hCT binding to live cells after 20 min co-incubation was 7.2±1.3×10^−9^ M (Figure 1E), confirming the expected difference in affinity between hCT and sCT under these experimental conditions [12], [19].

### sCT, but not hCT, stimulation leads to a prolonged cAMP production in CT_(a)_ receptor expressing cells

**Figure 2:**
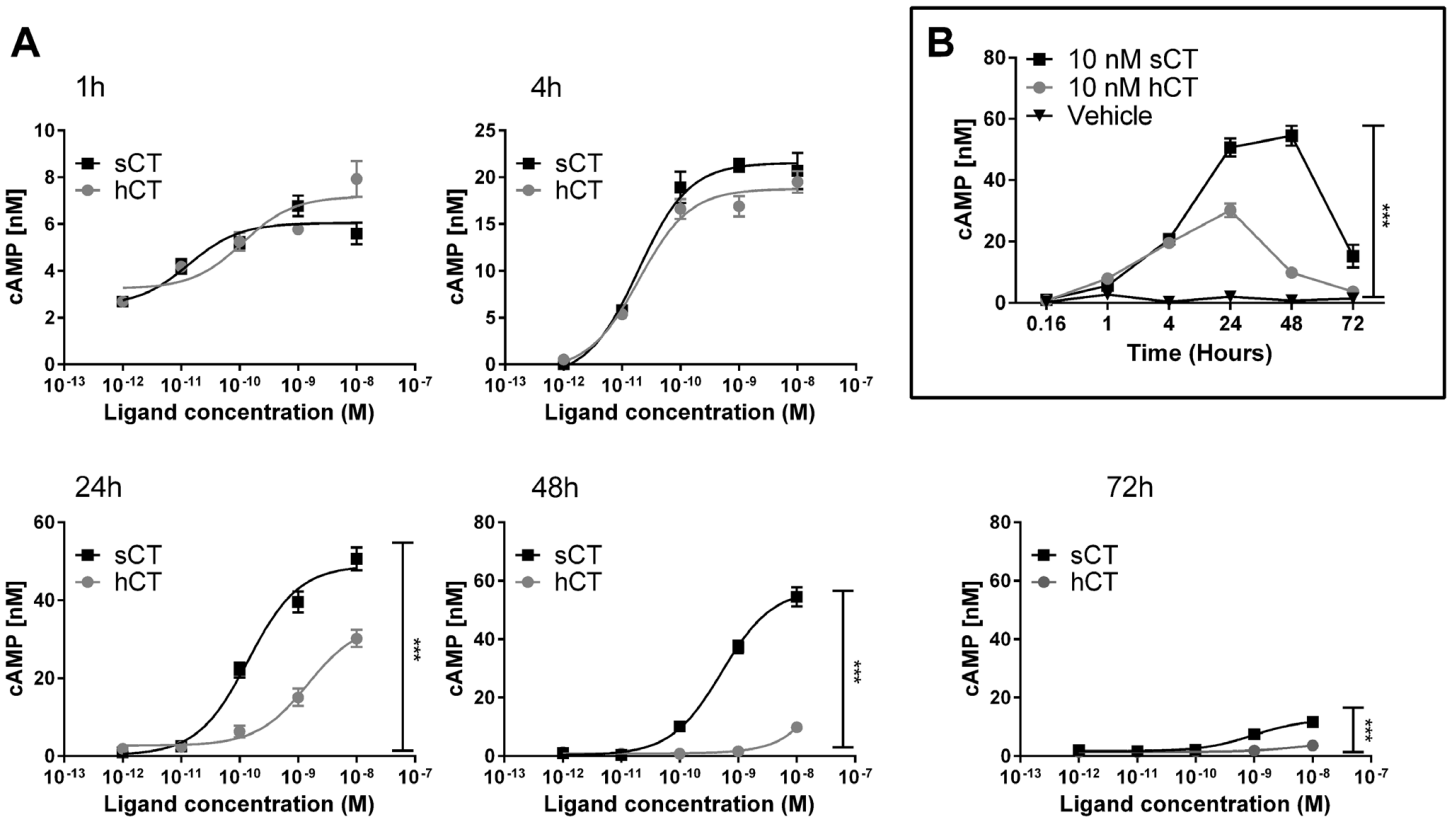
Difference in CT_(a)_R -mediated cAMP response by prolonged continuous hCT and sCT stimulation. A) cAMP production as a function of ligand concentration as U2OS CALCR cells were stimulated with sCT or hCT at a dose range of 10 pM to 10 nM for a prolonged period of time, ranging from 1, 4, 24, 48 to 72 hours. Ligands and medium were only added during the initiation of the experiment. Assays were conducted without IBMX in the medium to assess time dependent cAMP production. B) Single dose of 10 nM sCT, 10 nM hCT and Vehicle shown in (A) plotted as a function of time. Asterisk (*) indicate significant difference between AUC sCT and AUC hCT, p<0.05 was considered to be significant. *  = p<0.05, **  = p<0.01, ***  = p<0.001. Data are shown as mean ± SEM and representative of three individual conducted experiments using five replicates.

The distinct pattern of hCT and sCT binding to whole cells prompted further assessment of signaling responses to the two peptides. Initially, concentration-dependent cAMP production by sCT or hCT was assessed after 1, 4, 24, 48 and 72 hours (Figure 2A). sCT and hCT were close to equipotent after one and four hours, as shown in Figure 2A. After 24 and 48 hours a clear separation between sCT and hCT activation was observed, and after 72 hours sCT still induced a pronounced response, whereas hCT no longer had an effect. The difference over time is illustrated in Figure 2B, which demonstrates how 10 nM sCT maintains a prolonged cAMP production, peaking at 48 hours and still present at 72 hours, whereas the corresponding hCT-mediated cAMP peaks at 24 hours, and then dissipates, with no effect at 72 hours (P<0.001 for 24, 48 and 72 hours). This demonstrates a clear difference of the two peptides in prolonged stimulation of cAMP that is not observed with short-term stimulation (Figure 1A, 2A [1h], 2A [4h]). Comparable data were obtained using COS-7 CT_(a)_R cells (See Supporting Figure S1). These experiments were conducted with and without the PDE inhibitor IBMX, and while addition of IBMX did lead to markedly higher levels of cAMP in all the conditions, it did not alter the difference between sCT and hCT (See Supporting Figure S2).

### sCT, but not hCT stimulation, leads to prolonged β-arrestin recruitment and ERK1/2 activation in CT_(a)_ receptor expressing cells

**Figure 3:**
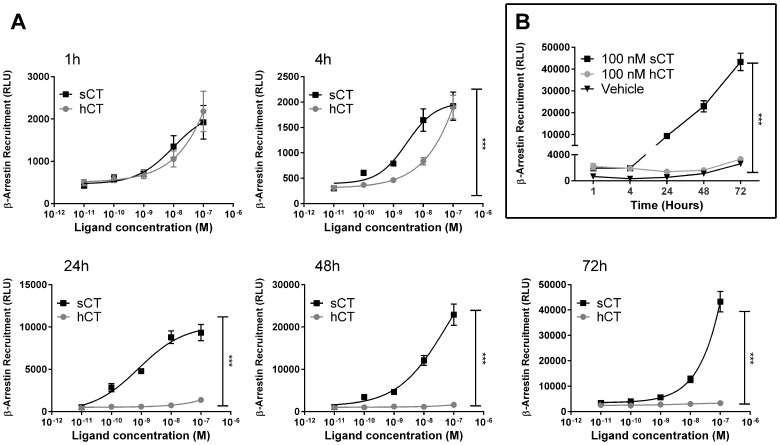
Difference in CT_(a)_R-mediated β-arrestin recruitment by prolonged continuous hCT and sCT stimulation. A) Data show β-arrestin recruitment as a function of ligand concentration. U2OS CALCR cells were stimulated with sCT or hCT at a dose range of 0.1 nM to 100 nM for a prolonged period of time, ranging from 1, 4, 24, 48 to 72 hours. Ligands and medium were only added at the experiment initiation. B) Single dose of 100 nM sCT, 100 nM hCT and Vehicle shown in (A) plotted as a function of time. Asterisk (*) indicate significant difference between AUC sCT and AUC hCT, p<0.05 was considered to be significant. *  = p<0.05, **  = p<0.01, ***  = p<0.001. Data are shown as mean ± SEM of 3 experiments performed with five replicates.

β-arrestin recruitment was also assessed using a prolonged response timeframe and sCT and hCT concentration-dependent responses after 1, 4, 24, 48 and 72 hours (Figure 3A) were determined using U2OS CALCR cells. sCT and hCT were close to equipotent after 1 hour as expected (See Figure 1). At 4 hours (Figure 3A [4h]) separation between sCT and hCT mediated β-arrestin recruitment could be observed. At 24, 48 and 72 hours the difference between sCT and hCT mediated β-arrestin recruitment was pronounced. The sCT response increased for each time point, whereas the hCT response was similar at 1, 4 or 24 hours but decreased to vehicle values at 48 and 72 hours. The difference over time for 100 nM ligand is illustrated in Figure 3B. These results correlate well with the cAMP induction data and demonstrate a clear difference in β-arrestin recruitment after prolonged stimulation by the two peptides compared to short-term stimulation (Figure 1B, 3A [1h]).

**Figure 4:**
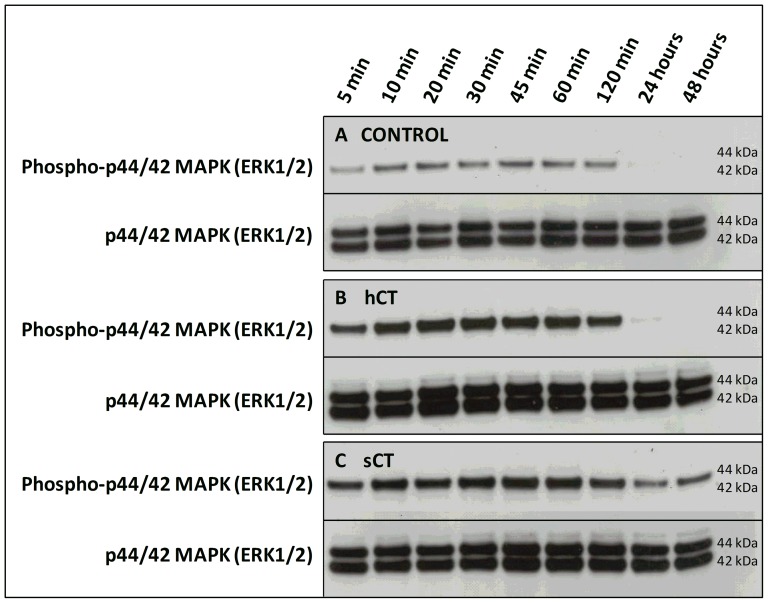
Prolonged ERK activation/phosphorylation in Cos-7 CT_(a)_R cells by sCT, but not hCT. Cos-7 CT_(a)_R Cells were stimulated for 5 min, 10 min, 30 min, 120 min, 24 hours or 48 hours with either Vehicle (A), 100 nM hCT (B) or 100 nM sCT (C). At each time point, the cells were lysed and protein content extracted. The protein extracts was then analyzed by western blotting investigating ligand-mediated phospho-ERK1/2 activation. Total ERK1/2 was used as a protein loading control.

As CT_(a)_R mediated β-arrestin recruitment is limited to the U2OS CALCR cell line, prolonged ERK1/2 activation by sCT and hCT was investigated in the Cos-7 CT_(a)_R cell line by western blotting (Figure 4). Cos-7 CT_(a)_R cells were treated with 100 nM hCT (Figure 4, Panel B), 100 nM sCT (Figure 4, Panel C) or vehicle (Figure 4, Panel A) for 5 min, 10 min, 30 min, 120 min, 24 hours or 48 hours. Both ligands increased the phosphorylation/activation of ERK1/2 in an equipotent manner from 5 to 120 min. However, ERK1/2 activation was only observed during prolonged sCT stimulation after 24 and 48 hours and not was present in the corresponding hCT stimulated Cos-7 CT_(a)_R cells or Control groups. This is consistent with the results from the prolonged β-arrestin recruitment response.

### Prolonged cAMP production and β-arrestin recruitment by sCT is only partially dependent of ligand occupancy of cell surface receptors

**Figure 5:**
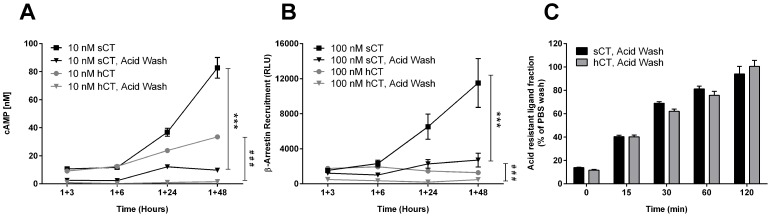
Acid wash decrease, but do not attenuate sCT mediated prolonged cAMP and β-arrestin signaling. Effects of acid wash on cAMP production (A) and β-arrestin recruitment (B). U2OS CALCR cells were initially stimulated for 60 min with sCT or hCT using U2OS CALCR cells, then acid washed for 2×2 min and cultured for another 3, 6, 24 or 48 hours in fresh ligand free culture medium, and then assayed at end incubation. In parallel, standard assay groups of sCT and hCT were included as controls. C) Internalization rate of ^125^I-sCT and ^125^I-hCT stimulated CT_(a)_ receptors in U2OS CALCR. Cells were pre-stimulated for 45 min at 4°C with 0.25 nM ^125^I-sCT or 0.25 nM ^125^I-hCT for ensure receptor occupancy, then incubating for an additional 0, 15, 30, 60 and 120 min at 37°C to assess internalization. Asterisk (*) indicates significant difference between AUC sCT, Not Washed and AUC sCT, Acid Wash. Asterisk (#) indicates significant difference between AUC hCT, Not Washed and AUC hCT, Acid Wash. p<0.05 was considered to be significant. *  = p<0.05, **  = p<0.01, ***  = p<0.001. cAMP data are shown as mean ± SEM and representative of three individual conducted experiments with five replicates. β-arrestin data are shown as mean ± SEM of 3 experiments performed with five replicates. Internalization data are shown as mean ± SEM and representative of two individual conducted experiments in triplicates.

To examine whether the prolonged response could be attributed to the continuous presence of unbound ligand or prolonged occupancy of cell surface receptors an acid wash experiment was conducted. After one hour of initial incubation by sCT or hCT, the cells were acid washed then re-incubated in fresh, ligand free culture for an additional 3, 6, 24 and 48 hours to assess the wash effect on the prolonged response using the same ligand concentration of 10 nM for cAMP and 100 nM for β-arrestin as shown in Figure 2B and 3B, respectively. Acid washed sCT stimulated cells demonstrated a significant decrease of the downstream effect for both cAMP production (Figure 5A) and β-arrestin recruitment (Figure 5B) compared to the non-washed controls, but was not completely abrogated and a prolonged response was still observed. In contrast, the hCT mediated cAMP production was completely abrogated by the acid wash (Figure 5A) and β-arrestin (Figure 5B) significantly lowered, though the observed time profile for β –arrestin recruitment was unaltered.

Measurement of internalization of the receptor following stimulation with either hCT or sCT (Figure 5C) did not demonstrate an apparent difference acid resistance, and the internalization level was equal at every time point measured. Internalization occurred at a steady incremental rate, and after 120 min all bound sCT and hCT was resistant to acid wash.

The retained prolonged sCT-mediated cAMP and β-arrestin signal following acid wash could possibly be explained by a sustained intracellular signal mediated by internalized constitutive active sCT-activated CT_(a)_ receptors, which were active for the full duration of the 48 hour experiment. This notion was supported by the internalization experiment as a substantial ligand mediated receptor internalization of ∼75–80% had already occurred at the onset of the acid wash step in the cAMP and β-arrestin experiments. As the acid wash removed ∼85–90% of all bound activity after 45 min of incubation with no expected internalization at 4°C (Figure 5C), roughly 10–15% of the observed cAMP and β-arrestin signal following the acid wash could be attributed to ligand still bound at the cell surface. The effect of washing with PBS was also investigated on β-arrestin recruitment (See Supporting Figure S3) and gave an intermediate response between non-washed and acid washed cells for both ligands.

### Continuous stimulation attenuates additional prolonged responses with an inverse correlation to ligand concentration

**Figure 6:**
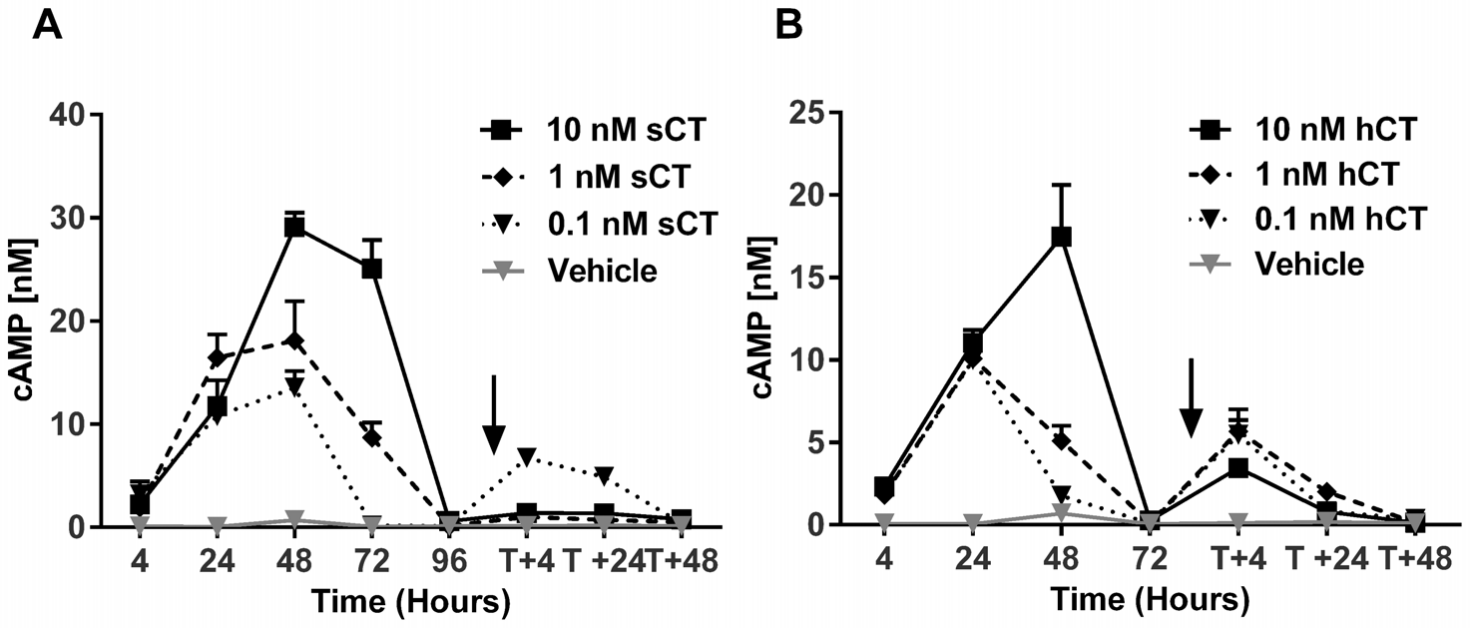
Continuous stimulation attenuates additional prolonged responses in inverse proportion to ligand concentration. U2OS CALCR cells were stimulated with sCT for 4, 24, 48, 72 or 96(A) or hCT for 4, 24, 48 or 72 hours (B) in a similar prolonged setup as demonstrated in Figure 2. In parallel, one set of cells was stimulated for 96h (sCT) or 72h (hCT), following which the culture medium was replaced with fresh medium with ligands and incubated for another 4, 24 or 48 hours. Ligand concentration sCT or hCT: 10 nM, 1 nM, 0.1 nM or vehicle. The vertical arrow indicates time of medium-ligand replacement.

As the acid wash and internalization experiments suggested the prolonged cAMP and β-arrestin response to originate partly from activated internalized receptors, we investigated whether the cells were responsive to a second round of ligand stimulation following an initial prolonged stimulation period. sCT (Figure 6A) and hCT (Figure 6B) were compared in a head-to-head setup similar to the one described in Figure 2. Based on results from Figure 2 the initial treatments were conducted for 96 and 72 hours for sCT and hCT, respectively, time-points where cAMP signaling had diminished to basal levels. The secondary stimulation was followed at three points, 4, 24 and 48 hours after reintroducing fresh ligand. At re-stimulation, we found an inverse relationship between the magnitude of the first prolonged response and the sustained response following the second treatment. This indicates that an increasing proportion of receptors were internalized or desensitized as a function of ligand concentration, a finding in line with other studies of GPCR regulation [25]. However, similar receptor reactivation responses between the two ligands was observed and thus, these responses do not appear important in the potency differences between sCT and hCT.

### Differences in ligand dissociation kinetics of ^125^I-sCT and ^125^I-hCT on membranes and living cells shed light on prolonged activation

**Figure 7:**
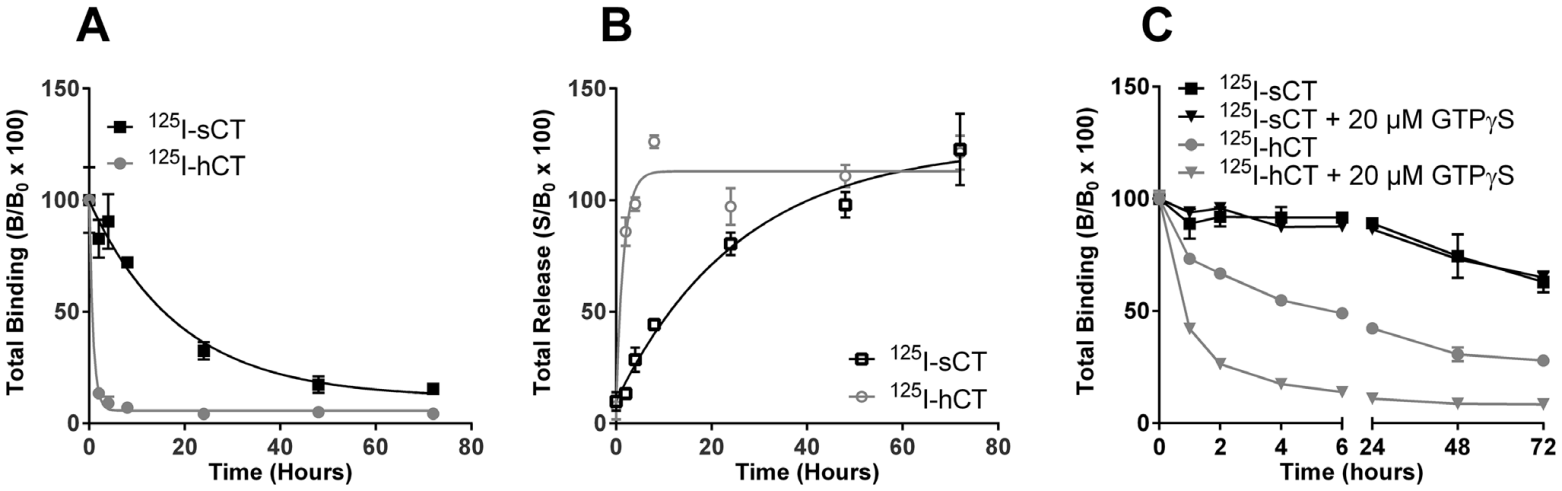
Prolonged ligand dissociation kinetics on membranes preparations and live cells. A) Total binding of ^125^I-sCT and ^125^I-hCT ligand bound to U2OS CALCR cells at different time points (0, 2, 4, 8, 24, 48 and 72 hours). B_0_ is total ^125^I-ligand bound to cells at t = 0 and B is ^125^I-ligand bound. B) Specific activity of ^125^I-sCT and ^125^I-hCT ligand released into the supernatant by cells in (B) at different time points (0, 2, 4, 8, 24, 48 and 72 hours). C) ^125^I-sCT and ^125^I-hCT ligand dissociation was investigated using isolated U2OS CALCR membrane preparations. The dissociation from the membranes was measured after 1, 2, 4, 8, 24, 48 and 72 hours with or without the presence of 20 μM GTPγS to determine G-protein dependency. B_0_ is total ^125^I-ligand bound to membranes at t = 0 and B is ^125^I-ligand bound.B_0_ is total ^125^I-ligand bound to cells at t = 0 and S is ^125^I-ligand in the medium. Live cell assays were conducted at 37°C and membrane assays were conducted at room temperature.

To elucidate the basis for the observed prolonged response and the emerging differences between membrane and live cell data, ligand dissociation from live cells and isolated membrane preparations was examined. The initial incubation time was 1 hour and 20 min of incubation for ^125^I-sCT and ^125^I-hCT, respectively, allowing maximum binding to be achieved (See Figure 1F). On live cells, ^125^I-sCT and ^125^I-hCT were both measured in the solubilized membrane (Figure 7A) and culture supernatant fractions (Figure 7B). sCT dissociated very slowly from the cells with a measured T_1/2_ of 13.2 hours with almost no dissociation within the first 4 hours. There was a subsequent progressive loss of radioactivity from the membrane fraction to the supernatant that was virtually complete by 48 hours, and this likely represents release of processed peptide following internalization of the receptor ligand complex. In contrast, hCT demonstrated rapid dissociation with an almost complete dissociation after ∼4 hours. The calculated T_1/2_ of 0.6 hours is virtually identical to the 36 min T_1/2_ value reported by Hilton *et al*. The corresponding pattern of sCT and hCT dissociation from membrane preparations (Figure 7C) correlated with the pattern of dissociation observed with live cells. Again, sCT demonstrated almost no dissociation during the first 24 hours and with 60% remaining bound after 72 hours giving an estimated T_1/2_ of 20.9 hours. Co-incubation with 20 μM GTPγS had no effect on sCT dissociation indicating G protein-independent dissociation at this concentration of nucleotide, thereby confirming observations by Hilton *et al*. In contrast to its rapid dissociation on live cells, hCT dissociated more slowly from membrane preparations with an estimated T_1/2_ of 2.1 hours and near complete dissociation after ∼48 hours. However, in contrast to sCT, hCT dissociated more readily from the CT_(a)_R when co-incubated with 20 μM GTPγS with a calculated T_1/2_ of 0.7 hours, a value close to the T_1/2_ observed with live cells. This suggests that, at the GTPγS concentration examined, hCT dissociation is dependent on G protein-interaction while sCT dissociation is G protein-independent, and this may contribute to the prolonged activation of the CT_(a)_R by sCT.

## Discussion

In this paper, we describe a possible novel mode of action in human CT_(a)_R signaling based on ligand-mediated differences in prolonged activation and possible cellular processing of the CT_(a)_R by two different CT_(a)_R ligands, sCT and hCT, which may explain the observed differences in potency between the two related peptides.

There were significant differences; most pronounced was the complete acid-wash induced loss of hCT mediated cAMP production, which was retained for sCT, though reduced in strength. The difference between sCT and hCT dissociation was also evident, as sCT demonstrated a very slow off rate from the CT_(a)_R in a G protein-independent manner, whereas hCT dissociated rapidly from the CT_(a)_R in a G protein-dependent manner.

These ligand-mediated differences were further underscored by measuring receptor activation over extended time periods where sCT induced a strong prolonged production of cAMP and β-arrestin recruitment for up to 72 hours. In contrast, hCT, while being close to equipotent during short-term stimulation, did not induce a continuously elevated β-arrestin response and the cAMP response was smaller in both signal strength and duration.

Interestingly, the physiological role of β-arrestin recruitment by the CT_(a)_R has not been studied, and while the continuous increase in β-arrestin recruitment by sCT-activated CT_(a)_Rs demonstrated in this paper is not fully understood, it indicates that β-arrestin may play a role in transmitting cellular activation signals, as opposed to just serving as a signal-attenuating arrestin [25], and further studies of this in a biological context are warranted.

It was noted that the EC_50_ between the cAMP and β-arrestin assays for both calcitonins were quite different; ∼5,000 fold. This is likely to reflect differences in both the affinity of ligands for the arrestin-bound state of the receptor and also the stimulus-response coupling efficiency for the two pathways, with the cAMP response being markedly amplified. Whether these differences can be translated to physiological *in vivo* output, remains to be elucidated.

Our findings that hCT and sCT are close to equipotent during short-term stimulation are in line with previous work on hCT and sCT activated cAMP production; two-fold difference in an overexpressing CT_(a)_ receptor cell line [12], equipotency in CT_(a)_ receptor-transfected cell lines [19] and a six-fold difference in T47D cancer cells [19].

Indications of prolonged activation of cAMP production and ligand binding by sCT have previously been published [3], [13], [14], and herein we strengthen this concept, as we observed prolonged activation of the CT_(a)_R using multiple outputs and obtained comparable results in two distinct cell lines.

It was expected that sCT would dissociate very slowly as this is consistent with previous studies [12], [26] and it has been shown that ^125^I-hCT is fully reversible on live cells [12]; data which are in line with our results on both live cells and membranes. Similarly, davalintide, an amylin/salmon calcitonin analogue dissociated very slowly from the amylin receptor on isolated membranes, whereas amylin was fully reversible [27]. Interestingly, the prolonged davalintide binding was manifest in a prolonged biological activity *in vivo*, supporting that this is not just an *in vitro* phenomenon, but a pharmacologically relevant activity and may explain at least part of the higher *in vivo* activity of sCT. Furthermore, the observed G protein-independency of sCT binding to CT_(a)_Rs is most likely linked to the slow off rate from the receptor, and may be a major component in the prolonged sCT response.

Another aspect of the prolonged response was elucidated by the acid wash experiment. The acid wash data suggest that a part of the prolonged sCT response is mediated by internalized receptors as both cAMP and β-arrestin responses were retained even when the receptor-bound ligand was stripped from the plasma membrane. In contrast, hCT mediated cAMP production was completely abrogated by the acid wash and a partial loss of the β-arrestin signal was observed. This may indicate that the prolonged response is only a feature associated with sCT.

Though, the hCT β-arrestin signal was sustained at low level for the full duration of the experiment, we hypothesize that this is a response related to the construction of the β-arrestin reporter, as it is not seen in the cAMP output studies. An observed partial loss in cAMP signal by PBS and acid wash was also reported by Michelangeli *et al* [13] after 30 min of sCT pre-incubation, though; their setup, time frame and purpose were different. In this relation, the wash data correlate well with the reactivation experiment showing that potent activation of the receptor eventually causes degradation/inactivation of the cell surface receptor with perhaps an increased intracellular activity of sCT stimulated receptors. The inability to re-stimulate is in line with published data demonstrating that the ligand activated CT_(a)_R is likely to be internalized and degraded post stimulation and dependent on protein synthesis rather than recycling to reestablish the receptor pool [28]–[30].

The internalization experiment demonstrated almost identical rate of internalization for sCT and hCT in terms of acid resistant ligand percentage with roughly 60% after 30 min, which is comparable to the T_½_ value of ∼45 min for CT receptor disappearance in T47D cells reported by others [28], [30].

The cell-based binding kinetics demonstrated a clear difference between sCT and hCT in terms of association and dissociation. Part of the difference can be explained by difference in binding kinetics, on both cells and membranes, but it is not the full story as hCT also displayed unusual association kinetics on live cells, which was not observed in the corresponding membrane experiments or for sCT at all. A speculative explanation of our combined association and wash data would be that hCT stimulated receptors, while having the same internalization rate as sCT stimulated receptors, are processed more rapidly compared to sCT stimulated receptors and we speculate that the latter remains constitutively active for a longer period of time.

However, it is difficult to compare hCT and sCT processing as dissociations rates are very different. Figure 7A–B provides a good indication of sCT ligand processing rate as dissociation is virtually non-existent, whereas the curve of hCT is a combination of dissociation and processing.

There are several known issues with hCT *in vitro*, such as lower potency due to an increased rate of fibrillation compared to sCT [31], [32], however, despite these differences hCT still has the same acute functional responses as sCT in our *in vitro* studies and hCT do generate a cAMP signaling that persists beyond the initial hours of incubation (Figure 2B). The described PBS (Supporting Figure S3) and acid wash studies (Figure 5) demonstrate that the lack of prolonged signal for hCT cannot be attributed to fibrillation of hCT. Hence, the washing data likely support the hypothesis that hCT and sCT are differentially biased in their regulation of CT_(a)_R function.

We propose that a possible explanation of the observed potency difference between sCT and hCT during the prolonged response is caused by the very slow receptor dissociation by sCT. This may cause internalized receptors to remain constitutively active even during the internalization/degradation process leading to an increase in cAMP signaling as well as an increase in the β-arrestin mediated signal cascade, including prolonged ERK1/2 activation.

An example of ligand differentiation in class B GPCR activation and regulation has been published [33]. The group demonstrated how two different parathyroid hormone (PTH) receptor ligands (PTH_1-34_ and PTHrP_1-36_) have a different MOA when it comes to receptor internalization routes and prolonged induction of cAMP production. Even though the PTH receptor is a fast acting receptor compared to the CT_(a)_R, it is possible that a similar difference between sCT and hCT can account for the suggested differences in sCT and hCT mediated receptor processing, although the time frame is vastly different (seconds vs. hours).

An additional contributing factor could be that sCT and hCT have slightly different binding interactions within the human CT_(a)_ receptor [2], [34]–[36] and these differences in receptor interaction may generate distinct ligand-mediated conformational changes of the activated receptor. This could contribute to the different observed downstream signaling by sCT and hCT, but this also remains to be elucidated.

There are few limitations to the study, mainly centered on what happens to the receptor once internalized, as we presently do not know to which compartment it is confirmed in the sCT-induced prolonged activation state. Furthermore, we do not know whether the intracellular processing of the ligand-receptor complex is different between the two ligands and studies focusing on inhibition of intracellular processing, i.e. in either lysosomes or endosomes would be of great interest to expand our findings. Finally, an intriguing point is to what extent the prolonged receptor activation elicits a biological response, i.e. is the inhibition of bone resorption by sCT prolonged compared to that by hCT [6], [37], and is the effect needed to get the anti-diabetic potential observed in the oral formulation of sCT applied in rats [16], [17].

### Conclusion

Based on our data, we suggest that a ligand-directed stimulus bias by sCT and hCT in the activation and regulation of the CT_(a)_R may exist and this is manifested in selective prolonged signaling across at least two distinct pathways. For sCT it may be mediated by constitutive active internalized receptors due to the inability to dissociate from the human CT_(a)_R, a mode of action not observed for the natural ligand, hCT. These data suggest that in addition to longer half-life, qualitatively very different responses to sCT and hCT are likely to occur *in vivo*.

## Supporting Information

Figure S1 **Direct effect on CT**_(a)_ **R-mediated cAMP response by prolonged hCT and sCT stimulation in Cos-7 CT**_(a)_ **R cells.** A) cAMP production as a function of ligand concentration in Cos-7 CT_(a)_R cells stimulated with sCT or hCT at a dose range of 10 pM to 10 nM for a prolonged period of time, ranging from 4, 8, 24, 48 to 72 hours. Ligands and medium were only added during the initiation of the experiment. Assays were conducted without IBMX in the medium to assess time dependent cAMP production. B) Single dose of 10 nM sCT, 10 nM hCT and Vehicle shown in (A) plotted as a function of time. Asterisk (*) indicate significant difference between AUC sCT and AUC hCT, p<0.05 was considered to be significant. *  = p<0.05, **  = p<0.01, ***  = p<0.001. Data are shown as mean ± SEM and representative of three individual conducted experiments with six replicates.
(TIF)

Figure S2 **CT**_(a)_ **R-mediated cAMP response by prolonged hCT and sCT stimulation in Cos-7 CT**_(a)_ **R cells with IBMX.** A) Data show cAMP production as a function of ligand concentration in Cos-7 CT_(a)_R cells stimulated with sCT or hCT at a dose range of 10 pM to 10 nM for a prolonged period of time, ranging from 4, 8, 24, 48 to 72 hours. Ligands and medium were only added during the initiation of the experiment. Assays were conducted with IBMX in the medium to assess time total cAMP accumulation. B) Single dose of 10 nM sCT, 10 nM hCT and Vehicle shown in (A) plotted as a function of time. Asterisk (*) indicate significant difference between AUC sCT and AUC hCT, p<0.05 was considered to be significant. *  = p<0.05, **  = p<0.01, ***  = p<0.001. Data are shown as mean ± SEM and representative of three individual conducted experiments with six replicates.
(TIF)

Figure S3 **Removal of unbound ligand by PBS wash does not alter the differences in calcitonin responses.** A) Beta-arrestin recruitment comparison between U2OS CALCR cells that were continuously stimulated for 3, 6, 24 or 48 hours with sCT and hCT. In parallel, cells were washed in PBS after one hour of initial ligand stimulation by sCT or hCT and then cultured for the remaining incubation period of 3, 6, 24 or 48 hours in fresh culture medium. Asterisk (*) indicate significant difference between AUC sCT and AUC hCT. B) Single dose of 100 nM sCT, 100 nM hCT, 100 nM sCT + Wash and 100 nM hCT + Wash illustrated in (A) plotted as a function of time. Asterisk (*) indicate significant difference between AUC sCT and AUC sCT Wash. Asterisk (#) indicate significant difference between AUC hCT and AUC hCT Wash, p<0.05 was considered to be significant. *  = p<0.05, **  = p<0.01, ***  = p<0.001.
(TIF)
