# Supplementary figures and images for: Prolonged Calcitonin Receptor Signaling by Salmon, but Not Human Calcitonin, Reveals Ligand Bias
Source: PLoS One. 2014 Mar 18;9(3):e92042. doi: 10.1371/journal.pone.0092042 (PMC3958426; doi:10.1371/journal.pone.0092042)

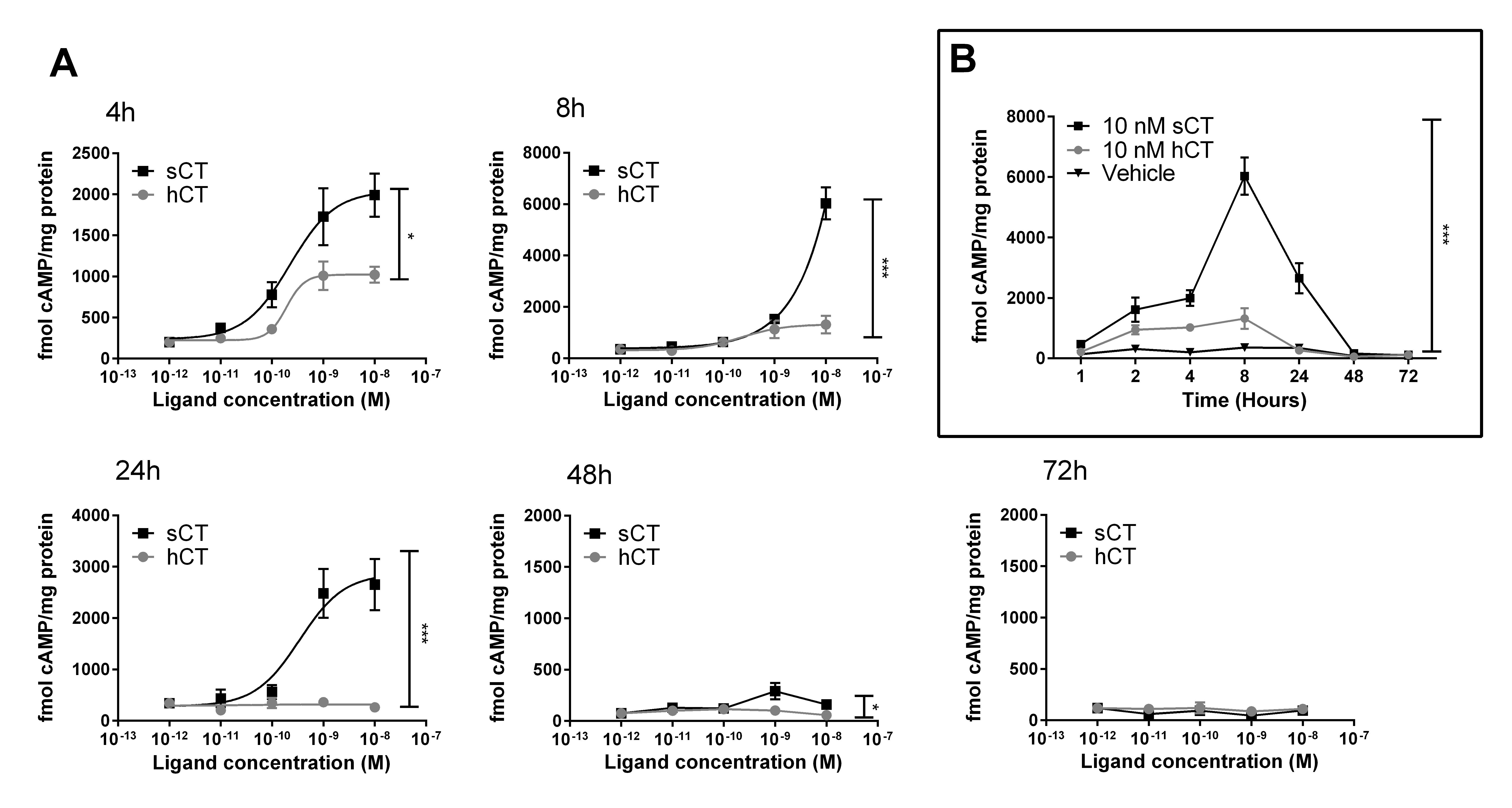

Supplement: Figure S1 — Direct effect on CT (a) R-mediated cAMP response by prolonged hCT and sCT stimulation in Cos-7 CT (a) R cells. A) cAMP production as a function of ligand concentration in Cos-7 CT(a)R cells stimulated with sCT or hCT at a dose range of 10 pM to 10 nM for a prolonged period of time, ranging from 4, 8, 24, 48 to 72 hours. Ligands and medium were only added during the initiation of the experiment. Assays were conducted without IBMX in the medium to assess time dependent cAMP production. B) Single dose of 10 nM sCT, 10 nM hCT and Vehicle shown in (A) plotted as a function of time. Asterisk (*) indicate significant difference between AUC sCT and AUC hCT, p<0.05 was considered to be significant. * = p<0.05, ** = p<0.01, *** = p<0.001. Data are shown as mean ± SEM and representative of three individual conducted experiments with six replicates. (TIF) [file pone.0092042.s001.tif]

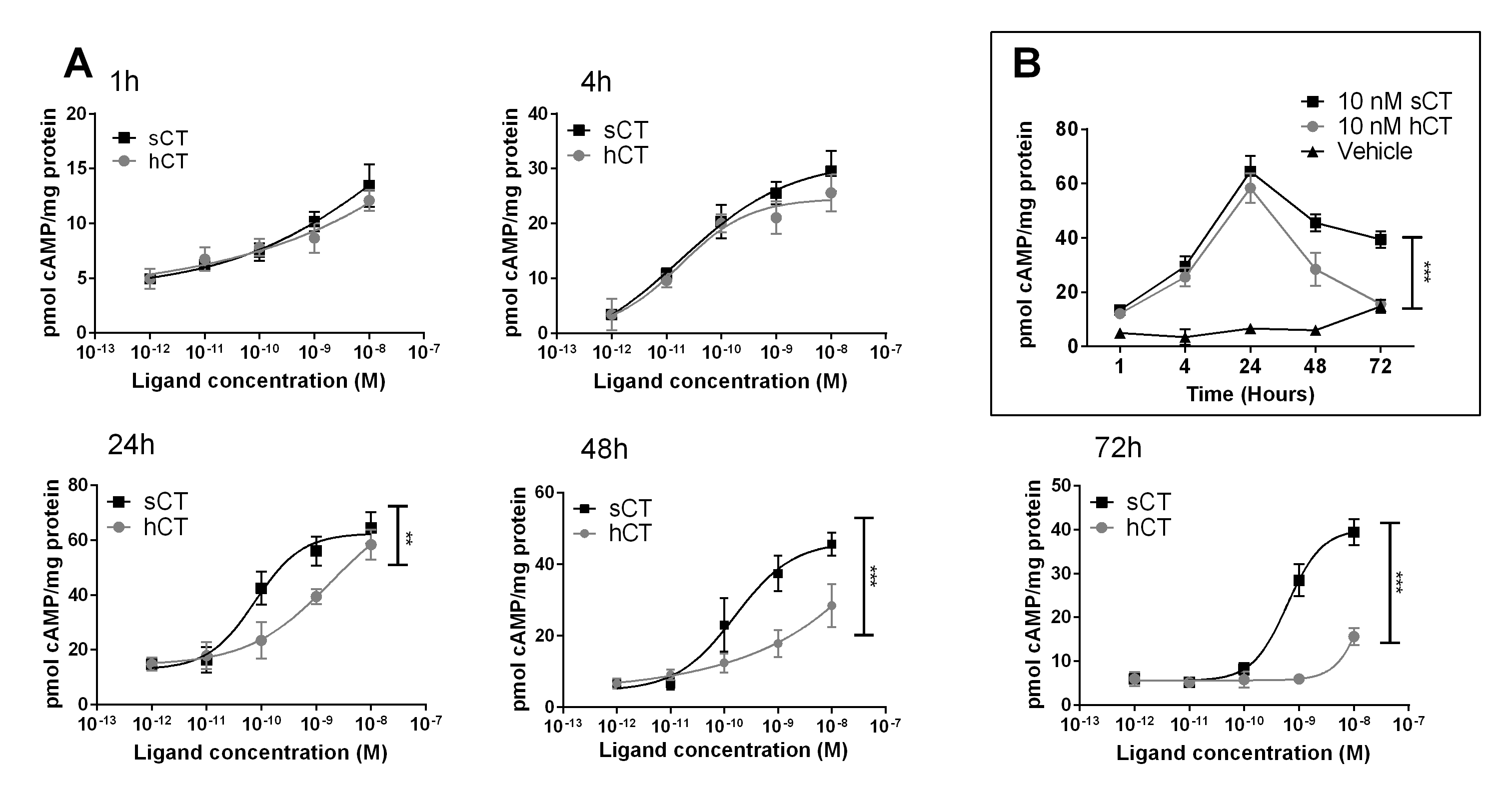

Supplement: Figure S2 — CT (a) R-mediated cAMP response by prolonged hCT and sCT stimulation in Cos-7 CT (a) R cells with IBMX. A) Data show cAMP production as a function of ligand concentration in Cos-7 CT(a)R cells stimulated with sCT or hCT at a dose range of 10 pM to 10 nM for a prolonged period of time, ranging from 4, 8, 24, 48 to 72 hours. Ligands and medium were only added during the initiation of the experiment. Assays were conducted with IBMX in the medium to assess time total cAMP accumulation. B) Single dose of 10 nM sCT, 10 nM hCT and Vehicle shown in (A) plotted as a function of time. Asterisk (*) indicate significant difference between AUC sCT and AUC hCT, p<0.05 was considered to be significant. * = p<0.05, ** = p<0.01, *** = p<0.001. Data are shown as mean ± SEM and representative of three individual conducted experiments with six replicates. (TIF) [file pone.0092042.s002.tif]

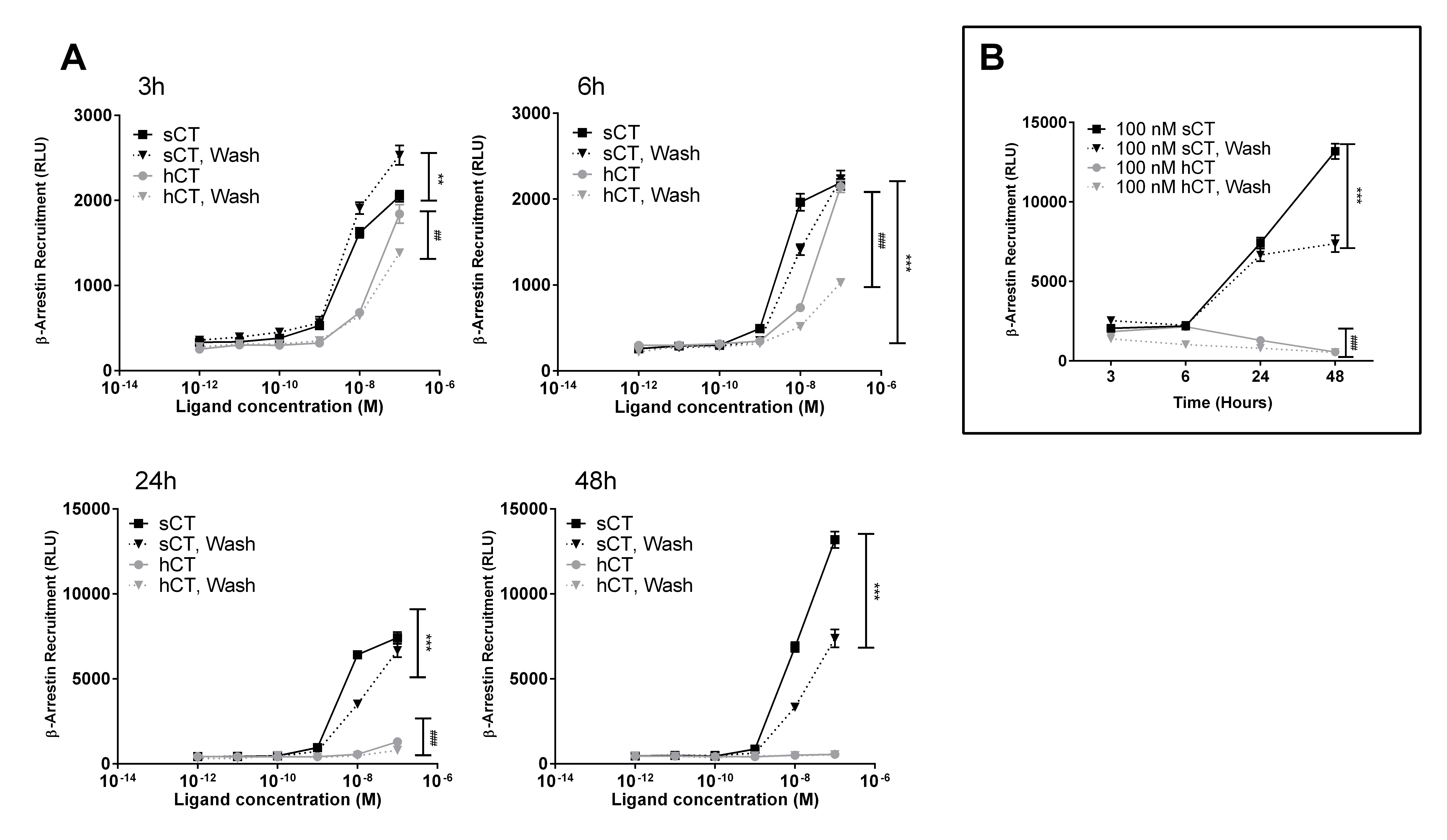

Supplement: Figure S3 — Removal of unbound ligand by PBS wash does not alter the differences in calcitonin responses. A) Beta-arrestin recruitment comparison between U2OS CALCR cells that were continuously stimulated for 3, 6, 24 or 48 hours with sCT and hCT. In parallel, cells were washed in PBS after one hour of initial ligand stimulation by sCT or hCT and then cultured for the remaining incubation period of 3, 6, 24 or 48 hours in fresh culture medium. Asterisk (*) indicate significant difference between AUC sCT and AUC hCT. B) Single dose of 100 nM sCT, 100 nM hCT, 100 nM sCT + Wash and 100 nM hCT + Wash illustrated in (A) plotted as a function of time. Asterisk (*) indicate significant difference between AUC sCT and AUC sCT Wash. Asterisk (#) indicate significant difference between AUC hCT and AUC hCT Wash, p<0.05 was considered to be significant. * = p<0.05, ** = p<0.01, *** = p<0.001. (TIF) [file pone.0092042.s003.tif]
